# Molecular cytogenetics as a clinical test for prognostic and predictive biomarkers in newly diagnosed ovarian cancer

**DOI:** 10.1186/1757-2215-6-2

**Published:** 2013-01-04

**Authors:** Shelly Gunn, Xavier Reveles, Korrie Weldon, Andres Barrera, Mariam Ishaque, Dale Taylor, Chris McCaskill, Jaeweon Kim, Rashmi Shah, Mansoor Mohammed, Todd Barry, Brianne Kaiser, Amita Patnaik, Anthony Tolcher

**Affiliations:** 1 Start Center for Cancer Care, San Antonio, TX, USA; 2 University of Texas Health Science Center School of Medicine, San Antonio, TX, USA; 3 PathCentral, Irvine, CA, USA

## Abstract

**Background:**

There is a clinical need for routinely available genomic biomarker testing in newly diagnosed ovarian cancer. In the current study we performed molecular cytogenetics using a validated array based comparative genomic hybridization (array CGH) assay to screen for the presence of predictive and prognostic biomarkers in archival diagnostic tissue from ovarian cancer patients. We hypothesized that biomarkers of high-risk disease would be detectable in tumor samples from patients with treatment refractory, advanced disease, and would be detected less frequently in tumor samples from patients with more favorable outcomes. In addition, we predicted that the use of a genome-wide copy number analysis (CNA) testing platform would enable us to identify novel potentially targetable chromosomal alterations of therapeutic significance in a percentage of cases.

**Methods:**

Formalin-fixed paraffin-embedded tissue (FFPE) tumor bank specimens were retrieved from the initial surgical resection for 18 ovarian cancer patients. Molecular cytogenetics was performed by array CGH for the detection of somatic chromosomal alterations associated with high-risk disease including amplifications of the CCNE1 and HER2 genes. Genomic risk stratification results were correlated with available clinical data. CGH data from each patient’s tumor genome was also surveyed for the presence of potentially targetable aberrations. Relevant therapeutic agents and open studies for investigational drugs were reported for each patient.

**Results:**

High-risk genomic alterations were identified in 12/18 (67%) of cases and all patients with high-risk markers had advanced, treatment refractory disease. Three tumors with minimal genomic changes had no high-risk markers and were from patients with Stage I/II disease that had been completely resected and under surveillance for recurrence. Eleven patients (61%) had at least one potentially targetable genomic alteration including CCNE1, HER2, KRAS gene amplifications, and somatic BRCA1 and/or BRCA2 gene deletions. Bi-allelic PTEN gene deletion was detected in one patient’s tumor.

**Conclusions:**

Clinical genomic profiling of ovarian tumors by array CGH augments pathologic grade and stage to help stratify newly diagnosed ovarian cancer into high and low-risk disease. This personalized genomic information can also help guide treatment planning and disease monitoring by identifying novel potentially targetable genomic alterations that can be used by clinicians to choose rational directed therapies for patients with chemo-resistant disease.

## Introduction

Ovarian cancer is a leading cause of cancer deaths in North American women. The majority of cases are diagnosed at an advanced stage, and therapeutic options are generally limited to aggressive surgical resection and adjuvant platinum-based chemotherapy regimens. Histologic subtype, tumor grade, and disease stage are currently used to stratify patients into high versus low-risk disease, and predict response to conventional therapy. Observed heterogeneity of disease course within these risk stratification groups has historically been attributed to the biology of each individual tumor genome, but until recently the underlying DNA alterations driving tumor behavior were not routinely detectable by the clinical laboratory.

It has been known for over two decades that HER2 overexpression is associated with poor survival in ovarian cancer and an estimated 10% of ovarian cancers are reported to be HER2 positive [1,2]. Curiously HER2 status is still not routinely evaluated as a prognostic/predictive marker in ovarian cancer even in advanced treatment refractory cases. This is particularly remarkable in light of the availability of not only FDA approved diagnostic testing, but also the increasing number of FDA approved anti-HER2 and anti-EGFR family therapies [3]. The low percentage of HER2 positive ovarian cancer cases may be among the reasons why routine HER2 testing is not performed, however NSCLC adenocarcinoma cases are increasingly sent for ALK testing even though the prevalence of ALK positivity is estimated to be only about 5% [4]. With approximately 200,000 ovarian cancer cases a year affecting women worldwide, an estimated 20,000 patients may have undetected HER2 positive ovarian cancer.

**Figure 1:**
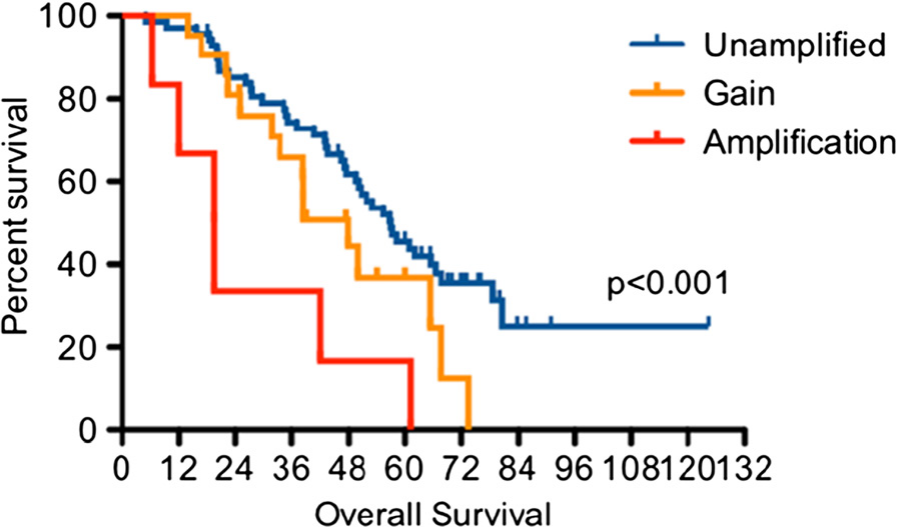
**CCNE1 copy number associated with patient outcome.** Adapted from Etemadmogham D, George J, Cowin PA, Cuillane C, Kansara M, et al. (2010).

More recently, amplicon-dependent expression of the cell cycle protein cyclin E has been described as a significant predictor of survival in advanced ovarian cancer [5,6]. Recurrent amplifications of the CCNE1 gene region on chromosome 19q, which encodes cyclin E, were reported in ovarian tumors by Etemadmoghadam and colleagues who used siRNA-mediated knockdown studies to show CCNE1 to be the amplicon driver of the oncogenic phenotype. They have subsequently validated the CCNE1 gene as a biomarker for chemoresistance in ovarian cancer [7]. CCNE1 gene expression is hypothesized to confer a post-treatment survival advantage to the tumor cells with high-level amplifications conferring a worse prognosis than low-level gains of the CCNE1 region (Figure 1). CCNE1 gene amplification is also a predictive therapeutic response biomarker for a growing number of preclinical and FDA approved targeted therapeutic options including BMS-387032, P1446A-05, flavopiridol, and seliciclib [8].

Somatic BRCA1 and BRCA2 gene alterations have been reported in sporadic epithelial ovarian cancers including loss of expression, somatic mutations, and LOH (deletions) [9]. Most BRCA gene studies in ovarian cancer have centered around the differential chemosensitivity profile known as “BRCAness syndrome” in selected patients with inherited BRCA1 and BRCA2 mutations who are reported to show enhanced sensitivity to platinum-based anticancer drugs [10]. However, clinicopathologic studies have shown most ovarian cancers with germline BRCA1 and BRCA2 mutations to be morphologically and clinically similar to sporadic ovarian cancer. These findings suggest that the loss of BRCA1 and BRCA2 gene function is important in ovarian carcinogenesis and BRCA genes drive ovarian tumor behavior whether the loss of gene function is caused by germline mutations, somatic alterations, or both. Therefore, detecting somatic alterations is of particular importance in the search for alternative ovarian cancer therapies because recent studies have shown that inhibitors of poly(ADP-ribose) polymerase (PARP) are potential targeted therapies for ovarian cancers with loss of BRCA1 and/or BRCA2 function [11].

The clinical availability of high-throughput genome wide copy number testing platforms and recently reported predictive/prognostic ovarian cancer biomarkers introduce a new era for risk stratification and treatment planning in ovarian cancer [12]. Biomarker identification has also fueled a growing awareness of increasing opportunities for novel targeted therapies [13]. These new high throughput testing methodologies, can detect multiple ovarian cancer biomarkers including focal amplifications of the HER2 and CCNE1 oncogenes, and somatic deletions of the BRCA1, BRCA2 genes in a single test. Many of these biomarkers can strongly predict disease course and response to therapy, but few have made their way into routine clinical use.

In order to develop a molecular cytogenetics testing strategy that would make genomic analysis of ovarian cancer biomarkers routinely available for our patients, we used clinically validated oligonucleotide and BAC array CGH assays to perform high throughput copy number analysis in archival ovarian tumor tissue from patients with known disease status. Our CGH tests provided a genome-wide survey of CNAs for the identification of known and novel predictive/prognostic biomarkers in each ovarian cancer patient’s tumor. As part of our study, we developed a reporting structure that would clearly and concisely communicate the actionable results to the clinician including prognostic biomarkers, and potential targeted therapeutic options.

### Molecular testing methods

#### Patient selection and tissue retrieval

Archival tumor samples from 18 cases of ovarian cancer with known disease status were retrieved from the START Cancer Center Tumor Bank. The tumor histologic subtypes included one clear cell, one granulosa, and 16 papillary serous carcinomas. Informed written consent was obtained from all patients and samples with accompanying case histories were de-identified for inclusion in the study.

#### DNA extraction from FFPE tissues

H&E slides were prepared with tumor areas visualized and circled by a pathologist. Targeted tissue areas were captured by slide scraping and following proteinase K digestion, a minimum of 2 μg DNA was isolated using the Promega Maxwell 16, with verification of high molecular weight DNA by agarose gel electrophoresis.

#### Array-based genomic analysis

Oligonucleotide array CGH analysis of chromosome copy number changes within the tumor genomes was performed for 14 cases at PathCentral, Inc. (Irvine, CA, USA) using the Agilent Human Genome CGH Microarray SurePrint G3, 8x60K custom array according to the manufacturer's instructions. BAC array CGH analysis was performed for four cases at Combimatrix Diagnostics, (Irvine CA, USA) using the DNAarray Tumor Profile 3300 clone array. For both platforms, an Oncogene ratio chart was generated for each case with a complete overview of all genes analyzed as an index of the fold change in activity (single copy gain and loss) between the control and test population. Normal ratio values typically ranged between 1.2 and 0.8. The choice of DNA platform was made depending on the quality and quantity of available DNA and both methods analyzed the same genomic biomarkers. Clinically relevant genes were selected from previously published data for solid tumors and a complete list of biomarkers can be found in Table 1[14].

**Table 1:** Genomic biomarkers analyzed by molecular cytogenetics

					
AKT1	CD44	FGFR3	MAP2K1	MYCN	RPS6KB1
AKT2	CDH1	FLT1	MAP2K4	NF1	SHH
AKT3	CDK4	FLT3	MAPK1	NF2	SMAD2
APC	CDKN2A	FMR1	MECOM	NFKBIA	SMAD4
AR	CDKN2C	HRAS	MDM1	NKX2-1	SOX2
ARID1A	CKS1B	IDH1	MDM2	NRAS	STK11
ATM	CTNNB1	IDH2	MDM4	PAK1	SUFU
BCL2	ECT2	IGF1R	MEN1	PBRM1	TNFAIP3
BIRC5	EGFR	INSR	MET	PDGFRA	TOP1
BRAF	ERBB2	JAG1	MGMT	PIK3CA	TOP2A
BRCA1	ERBB3	JAK2	MITF	PTEN	TP53
BRCA2	ERBB4	JUN	MLH1	PTPRD	TSC1
CACNA1E	FAM123B	KDM6A	MSH2	RAB25	TSC2
CCND1	FBXW7	KIT	MYC	RB1	VHL
CCNE1	FGFR1	KRAS	MYCL1	RET	WT1

90 clinically relevant oncogenes selected from a census of the published literature were analyzed by array CGH in the 18 cases of ovarian carcinoma included in this study.

High risk biomarkers were defined as: gain or amplification of the CCNE1 or HER2 genes, any high level (ratio > 3.0) oncogene amplification such as MYC or CCND1 among others, and/or PTEN gene deletion. Based on the genomic profiling results, each patient’s tumor genome was stratified as either **HIGH RISK GENOMIC MARKERS DETECTED or NO HIGH RISK GENOMIC MARKERS DETECTED** with the relevant high-risk markers listed in the report. Each patient’s tumor genome was also surveyed for the presence of other biomarker with potential therapeutic implications and reported as: **POTENTIAL THERAPEUTIC TARGETS IDENTIFIED or NO POTENTIAL THERAPEUTIC TARGETS IDENTIFIED**. In cases where potential therapeutic targets were identified, relevant therapeutic agents and open studies for investigational drugs were reported for each patient.

## Results

**Figure 2:**
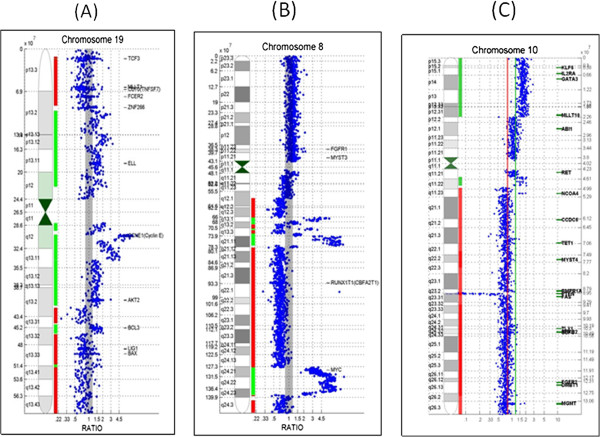
**Examples of oncogene amplifications detected by array CGH.** Individual chromosome ratio plots are shown with green representing amplified regions and red, the deleted regions. (**A**) Example of high-level CCNE1 gene amplification in case OC-04. (**B**) High level MYC gene amplification in case OC-08. (**C**) Bi-allelic PTEN deletion in case OC-18.

Prognostic genomic alterations were identified in 12/18 (67%) of cases, and all patients with high-risk biomarkers had advanced, treatment refractory disease. One patient (Case OC-13) was positive for HER2 gene amplification and four patients had CCNE1 gene amplifications or gains (Figure 2A). High level MYC gene amplifications were detected in four cases (Figure 2B). High level amplification of KRAS was detected in case OC-03 and high-level CCND2 in OC-10. The other two high-risk category patients had amplifications of CCND3 (Case OC-07) and PLAG1 genes (Case OC-10). Co-amplified genes were also prominent in the high-risk ovarian tumors including case OC-04 with high level CCNE1 amplification and co-amplification of CCND1, CCND3, MYC and ETV6 genes. A high-level CCND3 tumor (Case OC-07) showed co-amplification of MYC and AKT2; and a high-level MYC tumor (OC-08) was co-amplified for JAK2, FGFR3, and MYB. High-risk tumor genomes were defined by the highest amplification peak with other amplified genes designated as co-amplifications. One tumor with high-level MYC gene amplification also showed bi-allelic PTEN gene deletion (Figure 2C). Three tumors with minimal genomic changes and no high-risk markers were from patients with Stage I/II disease that had been completely resected and under surveillance for recurrence. Eleven patients (61%) had at least one potentially targetable genomic alteration including CCNE1, HER2, and KRAS gene amplifications. Somatic BRCA1 and/or BRCA2 gene deletions were detected in 9/18 (50%) of cases. Complete results are summarized and correlated with patient data in Table 2. Therapeutic targets and associated agents are summarized in Table 3.

**Table 2:** Prognostic biomarkers detected by array CGH in 18 cases of ovarian cancer correlated with clinical information

**Study #**	**High risk biomarkers**	**Disease summary and current status**
OC-1	MYC	Stage IIIC/serous; Locally advanced disease
OC-2	None detected	Stage IIIC/serous; Disease free under surveillance
OC-3	KRAS, CCND2,	Stage IIIC/serous; Non-resectable disease; progressive
OC-4	CCNE1, CCND1, CCND3, MYC, ETV6	Stage IIIC/clear cell; Progressive non-resectable disease
OC-5	None detected	Stage 1C/granulosa; Disease free under surveillance
OC-6	None detected	Stage IIA/serous; Disease free under surveillance
OC-7	CCND3, MYC, AKT2,	Stage IIIC/serous; Locally recurrent disease
OC-8	MYC, JAK2, FGFR3, MYB	Stage IIB/serous; Stable disease following surgery for recurrence
OC-9	CCNE1, MYC, ETV6	Stage IV at diagnosis; Metastatic to brain; deceased June 2012
OC-10	PLAG1	Stage IIIC/serous; Recurrent disease
OC-11	None detected	Stage IIIC/serous; Progressive disease
OC-12	None detected	Stage IIIC/serous; Metastatic disease
OC-13	ERBB2 (HER2)	Stage IV serous/Metastatic to bones and chest; Platinum resistant
OC-14	CCNE1	Stage III/serous; Progressive local recurrence
OC-15	None detected	Stage 1A/serous; Disease free under surveillance
OC-16	MYC	Stage IIIC/serous; Locally advanced disease
OC-17	PAK1	Stage IIIC/serous; Recurrent disease
OC-18	PTEN deletion	Stage IIIC/serous; Progressive non-resectable disease

Stage at diagnosis and histologic subtype are listed with most current disease status.

**Table 3:** Predictive biomarkers detected by array CGH with associated approved or experimental relevant therapeutics

**Biomarker**	**Positive cases**	**Description**	**Target class**	**Therapeutics**
CCNE1 Amp	N=4	Cyclin E1	Cell cycle inhibitor	BMS-387032, flavopiridol, seliciclib, P1446A-05
ERBB2 Amp	N=1	HER2	Kinase	Lapatinib, trastuzumab, neratinib
KRAS Amp	N=1	v-Ki-ras2	Kinase	PD 0325901, GSK1120212, AS703026, MSC1936369B, AZD6244, ARRY-438162
BRCA1 Del	N=7	Early onset breast cancer gene 1	Poly ADP Ribose Polymerase inhibitors (PARP)	AZD2281, ABT-888, MK4827
BRCA2 Del	N=3	Early onset breast cancer gene 2	Poly ADP Ribose Polymerase inhibitors (PARP)	AZD2281, ABT-888, MK4827
Bi-allelic PTEN Del	N=1		PI3K\AKT\mTOR inhibitors	Rapamycin

*Therapeutics table adapted from The Cancer Genome Atlas Network: Integrated genomic analyses of ovarian carcinoma [8].

**Amp = amplification; *Del* =deletion.

Eight tumors were positive for amplifications of the chromosome 3q26 region that has recently been identified as a significant region of amplification in ovarian tumors from patients with high risk disease and poor outcome. This region contains the MECOM, SnoN/SKiL, and ECT2 genes all of which have been implicated in ovarian cancer pathogenesis. All cases in this study with 3q26 amplification were high grade serous carcinomas [15].

## Discussion

The advent and recent availability of molecular cytogenetics in the clinical laboratory has added a new tool for genomic pathologists to use when evaluating solid tumors characterized by recurrent amplifications and deletions such as those of the brain, breast, and ovary [16]. We have developed a genome-wide, predictive and prognostic biomarker testing strategy for ovarian cancer that can be performed on a single platform using array CGH analysis of archival tumor DNA. Evaluation of diagnostic tumor tissue from 18 patients and correlation with current disease status revealed high-risk biomarkers to be positive in cases with advanced disease and not detectable in cases with more favorable outcomes. In addition, we identified alternative therapeutic targets in 11 patients with platinum resistant disease.

The use of array CGH as our testing platform allowed a genome-wide view of the chromosomal status of each tumor. Over half of the cases (N=10) were positive for somatic BRCA1 and/or BRCA2 gene deletions. All BRCA deleted cases were serous carcinomas with genome-wide chromosomal instability patterns reminiscent of those seen in triple negative basal-like breast cancers. There was also a high frequency of MYC and CCNE1 gene amplifications. This data is consistent with recently published integrated analysis of human breast cancers showing basal-like breast cancer to be genetically similar to serous ovarian carcinoma [17]. No tumors showed focal TP53 gene deletions in our study. However recent exome capture and sequencing on DNA isolated from 316 serous ovarian carcinomas revealed TP53 mutations in at least 96% of samples [8,18]. The absence of copy number changes involving TP53 in our study strongly suggests that intragenic point mutations are the primary mechanism by which the protein’s tumor suppressor function is lost in ovarian cancer. Future biomarker test development for ovarian cancer would ideally include a clinical assay for TP53 exome sequencing for detection of specific mutations with clinical prognostic and predictive value.

Three cases with advanced disease were negative for literature derived high-risk biomarkers, however all of these cases were positive for 3q26 gene amplifications. This region has recently been reported to be important for ovarian carcinogenesis and a recurrent region of gene amplification in ovarian cancer [15]. Two of the genes included in the region, MECOM and ECT2, have been associated with ovarian carcinogenesis. The MDS1 and EVI1 complex locus protein EVI1 (MECOM/EVI1) is a proto-oncogene which functions as a transcriptional regulator. EVI1 overexpression has been linked in human acute myelogenous leukemia (AML), myelodysplastic syndrome (MDS) and chronic myelogenous leukemia (CML). Immunostaining studies also show clear evidence of EVI-1 overexpression in some solid tumors including ovarian [19,20]. Arsenic trioxide (ATO) has been studied as a potential therapy for EVI1 expressing solid tumors but is not currently in clinical trials for ovarian cancer [21]. ECT2 encodes a transforming cell cycle regulator protein highly expressed in ovarian tumors [22,23]. Ovarian cancer and other solid tumor patients with positive test results for the EV-1 biomarker may be included in future ATO clinical trials in order to determine if their tumors show response to this rational directed therapy.

Molecular cytogenetic profiling of ovarian tumors by array CGH does not replace pathologic stratification of newly diagnosed ovarian cancer. The anatomic pathology report documenting grade and histologic subtype is still the cornerstone of the diagnostic and prognostic workup. However, genomic information about each patient’s tumor can augment traditional pathology findings to assist oncologists in the personalization of treatment planning and disease monitoring. At our institution, anatomic pathologists identify appropriate solid tumor cases for post-diagnostic prognostic marker testing by molecular cytogenetics at the time of diagnosis. Using this pro-active approach, the underlying DNA alterations driving tumor behavior are routinely reported to the oncologist by our clinical laboratory. This information enhances risk stratification and often identifies novel potentially targetable chromosomal alterations of therapeutic significance. In addition, DNA from the primary tumor is stored and can be used for future testing in the event of disease recurrence or metastases. As new prognostic and predictive markers are identified and become clinically available for ovarian cancer, molecular cytogenetics will become part of the routine diagnostic workup enabling oncologists to better predict disease course and choose targeted therapies based on the genomic profile of each individual’s tumor genome.

## Competing interests

The authors declare that they have no competing interests.

## Authors’ contributions

KW, CM, RS, TB carried out the molecular genetic studies; MM and JK provided analysis and reporting of CGH data; AB, MI, DT correlated CGH findings with clinical data; XR, BK coordinated the study; SG directed the study and drafted the manuscript; AP and AT correlated genomic findings with clinical trials; All authors read and approved the final manuscript.
